# Impact of eight weeks endurance training on biochemical parameters and obesity-induced oxidative stress in high fat diet-fed rats

**DOI:** 10.20463/jenb.2016.03.20.1.5

**Published:** 2016-03-31

**Authors:** Seyed Reza Emami, Mahvash Jafari, Rouhollah Haghshenas, Aliasghar Ravasi

**Affiliations:** 1 Department of Biochemistry, Faculty of Medicine, Baqiyatallah University of Medical Sciences, TehranIran; 2 Exercise Physiology Research Center, Baqiyatallah University of Medical Sciences, TehranIran; 3 Department of Human Science, Semnan University, SemnanIran; 4 Department of Exercise physiology, Faculty of Physical Education and Sports Science, University of Tehran, TehranIran

**Keywords:** High Fat diet, obesity, endurance training, oxidative stress, plasma, rat

## Abstract

**[Purpose]:**

High-fat diets (HFD) feeding is an important risk factor for obesity that is accompanied with metabolic syndrome. Appropriate exercise is recommended for obesity prevention. The molecular mechanisms and cellular pathways activated in response to HFD and exercise are not well understood. The purpose of this study was to investigate the effect of 8 weeks endurance training on some plasma biochemical parameters and oxidative stress in HFD induced obese rats.

**[Methods]:**

Twenty-eight male Wistar rats were randomly divided into 4 groups: the standard diet (SD) group, endurance training group with a standard diet (ESD), HFD group, and endurance training group with high-fat diet (EHFD). After 8 weeks, blood samples were taken by cardiac puncture and plasma were used for determination of biochemical parameters and oxidative stress biomarkers.

**[Results]:**

HFD significantly increased malondialdehyde level and decreased the activities of superoxide dismutase, catalase, and glutathione S-transferase and the content of glutathione in the plasma. HFD also increased activities of aspartate transaminase, alanine transaminase, lactate dehydrogenase, as well as levels of total cholesterol, triglyceride and low-density-lipoprotein-cholesterol. However, endurance training showed protective effect on changes in these parameters.

**[Conclusion]:**

These findings suggested that HFD alters the oxidant-antioxidant balance, as evidenced by reduction in the antioxidant enzymes activities and glutathione level and enhanced lipid peroxidation. Endurance training can be beneficial for the suppression of obesity-induced oxidative stress in HFD rats through modulating antioxidant defense system and reduces the risk of obesity-associated diseases.

## INTRODUCTION

Obesity, a disease of the twenty-first century, is a serious nutritional problem that is accompanied with heart disease, diabetes, cancer, inflammation and metabolic syndrome^1-3^. Its prevalence is increasing, with 2.1 billion overweight adults worldwide in 2013, as compared with 857 million in 1980^4^. In Iran, the prevalence of obesity in 2008 was 26.3% among 30-70 year olds^5^. Obesity induction may be by sedentary life style, neuroendocrine, dietary or genetic changes^6, 7^.

High-fat diets (HFD) feeding is an important risk factor for obesity, which can accelerate the overproduction of reactive oxygen species (ROS) by NADPH oxidase activation^3, 6, 8, 9^. Increased reactive oxygen species (ROS) can cause oxidative damage to nucleic acids, proteins and lipids, leading to disruptions in cellular homeostasis and aggravated metabolic syndrome features^1, 3, 10^. Previous studies have demonstrated that chronic consumption of a HFD induces the diminished superoxide dismutase (SOD), catalase (CAT) and glutathione (GSH) and increase of lipid peroxidation in humans and animals, all of which can lead to oxidative stress and finally cell death^1-3, 6, 10^.

Exercise has numerous health-related beneficial effects. It can normalize body weight, body fat, and markers of inflammation in mouse models of diet-induced obesity^3^. Physical exercise can counteract weight gain by increasing energy expenditure and reducing food intake via the increase in appetite-regulating hormones levels such as nesfatin-1 and PYY11. Several in vivo and in vitro animal and human studies showed that exercise increases whole-body oxygen consumption, the production of ROS, and lipid peroxidation. Regular physical activity leads to an increase in the activities of antioxidant enzymes^12-14^. However, the appropriate exercise in conjunction with dietary treatment for obesity prevention is recommended as a very important method for preventing metabolic syndrome^15-16^.

The molecular mechanisms and cellular pathways activated in response to HFD and exercise are not well understood. The ability to neutralize oxidant species differs in various high fat diets formulas and exercise models^15-17^. To our best knowledge, there are few reports on long-term effects of HFD and endurance training on antioxidant system in various tissues^15-16^. Therefore, the present study was designed to evaluate the activities of antioxidant enzymes such as SOD, CAT and glutathione S-transferase (GST), malondialdehyde (MDA) level as an important index of lipid peroxidation and GSH concentration, as well as biochemical parameters measurement in plasma after 8 weeks of exercise in the rat HFD model.

## METHODS

### Animals

Twenty-eight male Wistar rats weighting between 160 and 180 g were purchased from Pasteur Institute (Tehran, Iran) and acclimated for at least 1 week prior to experimental use. The animals were housed in normalized light-polyethylene cages in a room with 12/12 hours light/dark cycle at 22 ± 2°C and a relative humidity of 60 ± 5%. Water was available *ad libitum*. Rats were maintained in accordance with the Guidelines for the Care and Use of Laboratory Animals of Tehran University.

### Experimental design

Twenty-eight male Wistar rats were randomly divided into 4 groups, each comprising of 7 animals: the standard diet (SD) group, endurance training group with a standard diet (ESD), High-fat diet (HFD) group and endurance training group with high-fat diet (EHFD). The rats in the SD group were fed daily with a laboratory chow (containing 407 kcal/100 g total energy, 57% carbohydrate, 16% protein, and 25% fat based on percentage of total calories; Razi Institute, Iran). The rats in the HFD group were fed with HFD (containing 457kcal/100 g total energy, 37% carbohydrate, 13% protein, and 50% fat based on percentage of total calories; Razi Institute, Iran)^11, 18^. Each trained rat was exercised between 8 and 10 am, 5 days a week for 8 weeks. The rats progressively ran on a motor-driven rodent 4-channel treadmill from 15 min/day at 15 m/min speed, 0% slope, up to 60 min/day at 25 m/min speed, 0% slope. Animals from sedentary groups were placed for the same period on a turned-off treadmill.

### Body weight and the food intake

Body weight of the rats in each group was recorded daily. Food intake was estimated daily by differential weighting for each group of 7 rats and summed.

### Plasma preparation

Twenty-four hours after the last training session, rats in each group were anesthetized with diethyl ether after an overnight fasting. Blood samples were collected by cardiac puncture in heparin (0.2 mg per 1 ml of blood) as the anticoagulant and immediately centrifuged at 300×g for 15 min at 4°C. Plasma were removed and stored in 0.5 ml aliquots at –70oC freezer until biochemical analysis.

### Plasma antioxidant enzyme activities assay

The activity of SOD was determined using the method described by Winterbourn^19^, based on the ability of SOD to inhibit the reduction of nitroblue tetrazolium by superoxide. The amount of enzyme required to produce 50% inhibition was taken as 1 U and results were expressed as U/mg protein. CAT activity was measured in plasma using the method of Aebi^20^, by monitoring the decrease in absorbance at 240 nm in presence of hydrogen peroxide (H_2_O_2_) as the enzyme substrate. Specific activity was expressed as 1μmole H_2_O_2_ decomposed min^-1^ mg^-1^ protein. GST activity was determined by measuring the conjugation of 1-chloro-2, 4-dinitrobenzene (CDNB) with reduced glutathione that produced a dinitrophenylthioether, which was accompanied by an increase in absorbance at 340 nm^21^. The enzyme activity was expressed as μmol CDNB utilized/min/mg protein.

### Measurement of GSH content

GSH level was measured using Tietz^22^ method based on a continuous reduction of 5, 5′-dithiobis 2-nitrobenzoic acid to 5-thio-2-nitrobenzoic acid by catalytic amounts of reduced glutathione by catalytic amounts of GSH. The level of GSH was expressed as nmol/mg protein.

### Determination of lipid peroxidation

The end product of lipid peroxidation was estimated by measuring the level of MDA according to the method of Kei^23^, based on the formation of red pigment, generated by reaction of MDA with thiobarbituric acid. MDA concentration was expressed as nmol/mg protein.

### Protein assay

Protein concentration was estimated according to the method of Bradford using bovine serum albumin as a standard^24^.

### Plasma biochemical parameters

Plasma glucose, urea, uric acid and creatinine levels and activities of aspartate transaminase (AST), alanine transaminase (ALT), alkaline phosphatase (ALP), lactate dehydrogenase (LDH), creatin kinase (CK) and γ-glutamyl transferase (GGT) were measured using Pars azmoun Company kits (Tehran-Iran).

### Plasma lipid profile

Total cholesterol (TC), triglyceride (TG) and high-density- lipoprotein-cholesterol (HDL-C) concentrations were measured using Pars Azmoun Company kits (Tehran-Iran). Very low-density-lipoprotein (VLDL), low-density-lipoprotein- cholesterol (LDL-C) and atherogenic indices were calculated using the Friedwald equation as follows: VLDL= TG/5; LDL-C= TC – HDL-C– (TG/5).

### Statistical analysis

All calculations were performed using INSTAT statistical software version 3.3. For comparing between groups, analysis of variance (ANOVA) test was used following by Tukey post hoc multiple comparison test. Significance level was based on *P*< 0.05. Results were expressed as mean ± SEM of 7 different rats.

## RESULTS

### The body weight and food intake

The change in the body weight and food intake of rats in the experimental groups was depicted in Table 1. The body weight in the ESD and EHFD groups significantly decreased, as compared to the HFD group (*P*<0.01). The food intake in the HFD group significantly increased, as compared to the SD group; but significantly decreased in the EHFD group, as compared to the HFD group (*P*<0.05).

**Table 1.** Effect of 8 weeks endurance training and high-fat diet on body weight, food intake and plasma biochemical parameters in 4 groups of rats

Parameters	EHFD	HFD	ESD	SD
Weight (g)	259.28±10.15	306.84±7.32^#^	259.95±8.53	292.05±6.57
Food intake (g)	145.00±4.86	151.00±5.16^*^	119.43±5.77^†^	122.86±9.32
Glucose (mg/dl)	156.75±10.20^*^	165.13±5.52^**^	121.51±7.62^†^	125.53±7.43
Urea (mg/dl)	29.51±1.74	30.38±1.75	25.75±1.29	26.38±1.26
Creatinine (mg/dl)	0.525±0.034	0.609±0.041	0.501±0.059	0.536±0.028
AST (U/L)	38.76±1.68	46.56±2.17^**^,^#^	33.17±2.58	34.31±1.19
ALT (U/L)	24.44±1.48	30.31±2.09^**^,^#^	19.44±1.14	22.27±0.85
LDH (U/L)	142.86±8.22	179.62±10.75^*^,^#^	138.86±8.94	139.29±7.55
CK (U/L)	46.89±2.44	51.39±3.73	44.73±3.61	43.85±2.42
GGT (U/L)	41.32±3.59	45.02±2.39	34.85±4.41	36.54±2.12
ALP (U/L)	168.86±3.77	172.63±5.61	163.55±8.99	164.75±3.91

Values are expressed as mean±SEM (n=7).

^*^p<0.05 and ^**^p< 0. 01 vs. SD group; ^#^*p*<0.05 vs. ESD and EHFD group; ^†^*p*<0.05 vs. EHFD group. SD: standard diet group; ESD: endurance training plus standard diet; HFD: high-fat diet group; EHFD: endurance training plus high-fat diet; AST: aspartate transaminase; ALT: alanine transaminase; LDH: lactate dehydrogenase; CK: creatin kinase; GGT: γ-glutamyl transferase and ALP: alkaline phosphatase.

### Plasma biochemical parameters

The effect of HFD and 8 weeks endurance training on biochemical parameters in different groups were summarized in Table 1. The increased glucose level was observed in HFD and EHFD groups. HFD significantly increased the activities of plasma AST (*P*<0.01), ALT (*P*<0.01) and LDH (*P*<0.05), as compared with the SD group. These parameters in the ESD and EHFD groups significantly decreased, as compared to the HFD group (*P*<0.05). There were no significant changes in urea and creatinine levels and activities of CK, ALP and GGT in different groups.

### Plasma lipid profile

Table 2 showed that the effect of 8 endurance training on plasma lipid profile in different groups. TG, TC, VLDL and LDL-C levels in HFD group were higher than SD group. These parameters in EHFD group were significantly lower than HFD group. There were no significant changes in HDL level, LDL to HDL and TC to HDL ratios in different groups. MDA level was increased by 25.69 and 8.94% in the HFD and EHFD groups in comparison to the SD group, respectively. MDA level in the ESD (*P*<0.001) and EHFD (*P*<0.05) groups significantly decreased, as compared to the HFD group.

**Table 2.** Effect of 8 weeks endurance training and high-fat diet on plasma lipid profile changes among 4 groups of rats.

				
TG (mg/dl)	65.5±3.61	88.38±3.92^*^,^#^	60.5±3.61	66.25±9.06
TC (mg/dl)	83.28±2.31	95.13±2.04^**^,^#^	72.25±3.11^†^	79.5±3.82
VLDL (mg/dl)	13.12±0.716	17.68±0.784^*^,^#^	12.11±0.722	13.25±1.81
LDL-C (mg/dl)	65.25±1.85	86.93±3.66^**^,^#^	51.73±4.55^†^	62.94±4.92
HDL-C (mg/dl)	31.13±2.14	25.88±2.79	32.63±2.48	29.81±3.85
L-DL-C/HDL-C (mg/dl)	2.09±0.392	3.36±0.443	1.59±0.651	2.11±0.244
TC/HDL-C (mg/dl)	2.68±0.408	3.68±0.276	2.21±0.475	2.67±0.375
MDA (nmol/mg protein)	2.09±0.074	2.42±0.101^**^,^#^	1.77±0.067^†^	1.93±0.071

Values are expressed as mean±SEM (n=7).

^*^p<0.05 and ^**^p< 0. 01 vs. SD group; ^#^*P*<0.05 vs. ESD and EHFD group; ^†^*P*<0.05 vs. EHFD group. SD: standard diet group; ESD: endurance training plus standard diet; HFD: high-fat diet group; EHFD: endurance training plus high-fat diet; TC: total cholesterol; TG: triglyceride; HDL-C: high-density-lipoprotein-cholesterol; VLDL: very low-density-lipoprotein, LDL-C: low-density-lipoprotein-cholesterol and malondialdehyde (MDA).

### Plasma antioxidant levels

**Figure 1.**
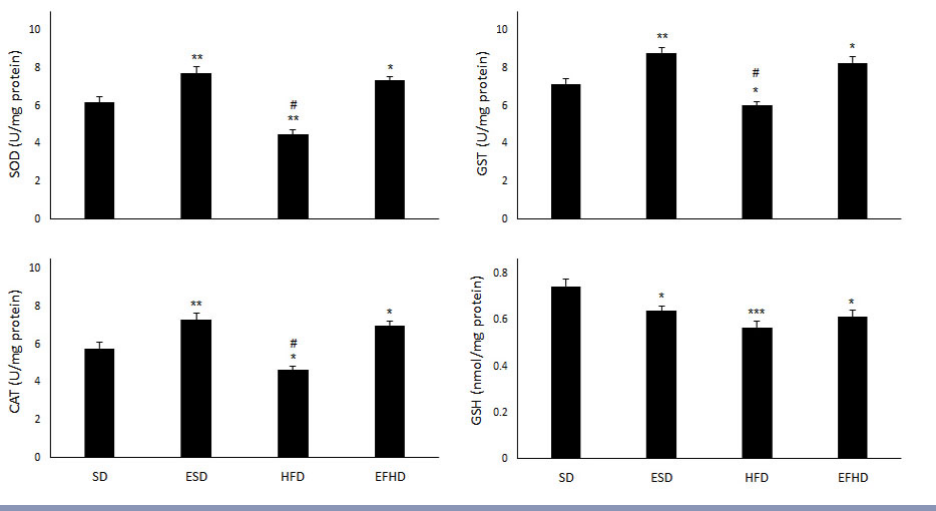
Effect of 8 weeks endurance training and high-fat diet on superoxide dismutase (SOD), catalase (CAT) and glutathione S-transferase (GST) activities and glutathione (GSH) level in four 4 groups of rats. Values are expressed as mean±SEM (n=7). ^*^p<0.05, ^**^p< 0. 01 and ^***^p< 0. 001 vs. SD group; ^#^*P*<0.05 vs. ESD and EHFD group. SD: standard diet group; ESD: endurance training plus standard diet; HFD: high-fat diet group; EHFD: endurance training plus high-fat diet.

Figs. 1 showed the alteration of SOD, CAT and GST activities and GSH content in the experimental groups. SOD, CAT and GST activities in plasma were decreased in the HFD group after 8 weeks, while these enzymes were increased in the ESD and EHFD groups, as compared with the SD and HFD groups. GSH level was decreased by 14.27, 23.66 and 17.77% in the ESD, HFD and EHFD groups, respectively.

## DISCUSSION

Consumption of HFD in rats is a useful model of putative effects of dietary fat in humans^2, 6, 25^. In the present study, HFD feeding for 8 weeks induced obesity in rats. The body weight and food intake of Wistar rats fed the HFD were significantly increased and the endurance training suppressed these effects. This significant lower body weight in trained rats may be due to the reduction of the amount of adipose tissue resulting in decreased generation of sex hormones, glucose, leptin^12^; the increase appetite-suppressing neuropeptide hormones levels such as nesfatin-1 and PYY levels, and negative energy and fat balance linked with increased energy expenditure and fat oxidation during the exercise^11, 17, 26^. However, Mohammadi et al showed that the increased body weight of rabbits by 8 weeks of high cholesterol diet feeding was not altered under chronic exercise^16^.

HFD-induced obesity induces the production of ROS and oxidative stress^6, 15^. A set of endogenous antioxidant enzymes such as SOD and CAT play an important role in the elimination of ROS and protect cells against the deleterious effects of oxidative stress^6^. SOD converts the superoxide anion generated by NADPH oxidase into oxygen and hydrogen peroxide (H_2_O_2_) and CAT catalyzes the conversion of H_2_O_2_ to water and oxygen^15, 27^. In this study, HFD decreased the SOD and CAT activities in plasma rat. The decrease in SOD activity could be due to feedback inhibition or oxidative inactivation of enzyme proteins due to excess ROS generation and the decreased de novo synthesis of SOD proteins^15, 28^. In addition, the depletion of SOD activity increases the endogenous superoxide anion, which inhibits CAT activity and leads to accumulation of H_2_O_2_ and the induction of oxidative stress. The present result is also consistent with several others showing that SOD and CAT activities were decreased in various tissues after HFD feeding^1, 2, 6, 8, 27-28^. However, previous studies have reported the increased SOD and CAT activities in rat liver and erythrocytes following feeding of HFD for 4 and 9 weeks^29-30^. Our findings showed that the activities of SOD and CAT were significantly increased in both exercise groups after 8 weeks. The enhanced activity of antioxidant enzymes in rats during exercise training is indicative of the capability to develop a compensatory mechanism to oxidative stress in tissue by means of an adaptation of the antioxidant and repair systems^12, 16, 31^. Numerous studies have shown that antioxidant enzymes activities were increased in blood or in tissues of animals and humans after aerobic exercise^14-16^. However, a study showed that exercise reduced the CAT activity after 4 weeks in rats^32^. The response of training on oxidative stress would appear different according to exercise type, duration, intensity, volume and duration, type of animal and method of assessment^16, 31^.

GST plays a key role in cellular detoxification of ROS by conjugation with the GSH and protects tissues from oxidative stress. GST activity can reflect the antioxidant capacity of the body^21, 33^. In present study, the GST activity was decreased in rats fed HFD, while it was increased in both exercise groups. The inhibition of GST activity in HFD-treated rats may be due to direct binding of toxic intermediates to essential sulfhydryl groups of these enzymes, inactivation of enzyme by increased ROS and organic peroxides and depletion of GSH, which is essential for GST activity^33^. Several studies reported decreased GST activity^2, 6^, others reported increases^8^, while others found no significant differences in liver after feeding of HFD^9^. This inconsistency of results may be a reflection of differences in type, percent and time consumption of fat diet, the animal type, breed and species and tissue type. Vukovic et al showed that HFD significantly reduced liver GST activity and physical activity did not induce significant changes in enzyme activity in rats that received HFD^32^.

Lipid peroxidation is one of the molecular mechanisms involved in obesity-induced toxicity. MDA is one of the end products of lipid peroxidation in the cell membrane, which is used as a marker of tissue oxidative stress^6, 10, 34^. In present study, MDA level was increased in HFD group without significant change in ESD and SHFD groups, as compared to SD group. HFD-induced obesity can cause increased lipid peroxidation and induce cell injury. These results are in agreement with the reports showing that HFD and exercise elevates lipid peroxidation products in animals^15, 16, 32^. However, a study in rats reported no change in MDA level in response to HFD and physical activity^34^.

GSH is a powerful non-enzymatic antioxidant in the cells that can directly scavenge ROS or act as a substrate for GST during the detoxification of hydrogen peroxide and lipid hydroperoxides. Its depletion is considered as an important biomarker of oxidative stress in animals and human^8, 25, 28, 35^. A significant depletion of GSH was noted in the present study in ESD, HFD and EHFD groups, as compared to the SD group. There were no significant differences in the GSH levels between HFD and exercise groups. Diminished GSH content in plasma of rats may be due to increased utilization of GSH for conjugation and/or participation of GSH as an antioxidant in neutralizing free radicals. Depletion of GSH leads to the production of oxidized GSH (GSSG) and finally decreases the ratio of GSH/GSSG in tissues of rats, which is indicative of oxidative stress^7, 25^. GSH is synthesized in the liver cells and then distributed through plasma to different organs. Diminished liver GSH reserve can also decrease plasma GSH^33^. Our result is in agreement with previous studies that have shown the effect of HFD and exercise on GSH level in different tissues^2, 10, 25, 32^. However, Jodynis-Liebert and Murias showed that HFD feeding after 4 weeks did not alter the GSH content in rat liver^36^.

The assay of AST, ALT, and LDH activities are used routinely for clinical diagnosis of disease and damage to the structural integrity of the liver^25, 33^. The high levels of these parameters in our study are attributed to fatty liver induced by HFD^37^. The increase of ROS and lipid peroxidation induced by HFD lead to an increased permeability of liver and the release of liver enzymes into the plasma^33, 38^. Several studies have reported that HFD caused degeneration in hepatocytes and changes of liver enzymes and lipid metabolism^25, 27, 37^. In this study, plasma AST, ALT and LDH activities were returned to normal levels in both exercise groups, suggesting the amelioration of fatty liver. This decrease may be the consequence of prevention of liver damage by the antioxidant potential of the exercise^39^. In this study, the significant increase in glucose in HFD fed animals can be due to defective insulin synthesis, decreased insulin efficiency, insulin resistance, decreased adiponectin hormone level and Na^+^-K^+^-ATPase activity^27, 35, 37^. It indicated that HFD leads to insulin resistance through oxidative stress^30^. Exercise training significantly reduced the increased blood glucose levels due to the increased adiponectin level and insulin sensitivity^12, 27^.

Obesity and hyperlipidemia are the major risk factors for cardiovascular diseases. Excess of LDL-C promotes the formation of atherosclerotic plaque^39^. In present study, the plasma levels of TC, TG, VLDL and LDL-C in the HFD group were increased due to increase in both de novo synthesis and intestinal absorption of cholesterol. HFD induces the production of ROS, which react with lipoproteins to produce oxidation states, thus diminishing the cellular uptake of lipids from the blood^35, 37^. These results are similar to results of other studies^6, 27, 38.^ However, these parameters were decreased in training groups when compared with the HFD group. The decrease of lipids may be caused by the inhibition of impaired lipid digestion and absorption, improvement in glucose and lipid metabolism, enhancement of insulin sensitivity, increased antioxidant defense, and down-regulation of lipogenic enzymes^27^. Several studies have demonstrated that exercise decreases blood lipid and lipoprotein levels lipids in humans and rats^7, 16^.

## CONCLUSION

These findings suggested that HFD feeding induces oxidative stress and disturbances in plasma hepatocellular enzymes and lipid levels. Endurance training can be beneficial for the suppression of obesity-induced oxidative stress in HFD rats through improved activity of antioxidant enzymes and decreased lipid peroxidation, and thereby modulate the obesity-related tissue damage.
